# Untapped potential of gut microbiome for hypertension management

**DOI:** 10.1080/19490976.2024.2356278

**Published:** 2024-06-02

**Authors:** Kan Gao, Pu Xiu Wang, Xue Mei, Tao Yang, Kai Yu

**Affiliations:** a Department of Pharmacy, The First Hospital of China Medical University, Shenyang, Liaoning, China; b School of Pharmacy, Institute of Materia Medica, North Sichuan Medical College, Nanchang, Sichuan, China; c Department of Physiology and Pharmacology, Center for Hypertension and Precision Medicine, College of Medicine and Life Sciences, The University of Toledo, Toledo, OH, USA; d Department of General Practice, The First Hospital of China Medical University, Shenyang, Liaoning, China

**Keywords:** Gut microbiota, hypertension, metabolite, antihypertensive medications, machine learning

## Abstract

The gut microbiota has been shown to be associated with a range of illnesses and disorders, including hypertension, which is recognized as the primary factor contributing to the development of serious cardiovascular diseases. In this review, we conducted a comprehensive analysis of the progression of the research domain pertaining to gut microbiota and hypertension. Our primary emphasis was on the interplay between gut microbiota and blood pressure that are mediated by host and gut microbiota-derived metabolites. Additionally, we elaborate the reciprocal communication between gut microbiota and antihypertensive drugs, and its influence on the blood pressure of the host. The field of computer science has seen rapid progress with its great potential in the application in biomedical sciences, we prompt an exploration of the use of microbiome databases and artificial intelligence in the realm of high blood pressure prediction and prevention. We propose the use of gut microbiota as potential biomarkers in the context of hypertension prevention and therapy.

Hypertension is a severe, chronic condition that increases the morbidity and mortality associated with cardiovascular diseases. The Global Burden of Disease (GBD) Study demonstrates that hypertension ranks first among modifiable risk factors that contribute to cardiovascular disorder burden.^1^ In 2019, high blood pressure was responsible for 53% of deaths from ischemic heart disease and 53% of stroke deaths. According to the data from the World Health Organization (WHO), approximately 33% of adults aged 30–79 are affected by hypertension. The total number of people with hypertension is increasing continuously, which doubled from 650 million in 1990 to 1.3 billion in 2019. According to a WHO statement, if even half of those with high blood pressure could have their condition under control by 2050, this would prevent 76 million deaths, 120 million strokes, 79 million heart attacks, and 17 million cases of heart failure.^2^

Many factors contribute to hypertension, including genetic and environmental factors,^3–5^ like pollution, cold temperatures and extreme altitude elevation, and personal environmental exposures, such as high sodium and low potassium diet, overweight or obesity, alcohol consumption, tobacco smoking and physical inactivity. The interaction between these factors is complex and incompletely understood. Recent research indicates that the human intestinal microbiota plays an important role in the occurrence and development of high blood pressure. In addition, there is a growing interest in new therapeutic methods and prevention strategies for manipulating the intestinal microbiota to improve the blood pressure of the host.

## 1. Characteristics of gut microbiota in healthy individuals

The human gastrointestinal tract harbors more microorganisms than the total number of human cells. These microorganisms contain around 100 times the number of genes than that found in the human genome, making them the “human second genome” that affects overall host health. Collectively, these microorganisms are known as the gut microbiota, which is composed of fungi, viruses, archaea, protists, and dominant bacteria.

The gut microbiota is primarily composed of six phyla, namely *Bacteroidetes*, *Firmicutes*, *Actinobacteria*, *Proteobacteria*, *Verrucomicrobia*, and *Fusobacteria*. Among these, the phyla *Firmicutes* and *Bacteroidetes* usually account for greater than 90% of total bacteria.^6^ The composition of the gut microbiota significantly varies across different intestinal anatomical segments.^7^ The quantity of microorganisms inhabiting distinct regions of the intestine differs considerably.^7^ The colon and distal ileum are the regions with the highest microbial counts per gram of excrement, with 10^11^ and 10^7–8^ cells, respectively. The content of microorganisms in the proximal ileum and jejunum is relatively low, with only 10^2–3^ cells per gram of feces.^8^

Under normal conditions, the intestinal microbiota contributes to the host homeostasis from several aspects.^9^ 1) it takes part in the nutrient absorption and the energy metabolism of human body. The gut microbiome ferments the polysaccharides into absorbable monosaccharides, and participates in the essential amino acids and vitamin synthesis and ion absorption. The process of fermentation also generates short-chain fatty acids (SCFAs), mainly including acetate, propionate, and butyrate. The metabolism of SCFAs and bile acids (BAs) provides energy and nutrients not only for bacterial growth and proliferation but also for the host. 2) it regulates host immune responses. The normal intestinal microbiota is the first line of defense against pathogens and toxins, working together with the host immune system to protect the body from diseases. 3) it protects the intestinal epithelial barrier function. A healthy microbiota prevents pathogen colonization through barrier effects and interacts directly with intestinal epithelial cells, leading to normal protective responses in the host, and initiating appropriate inflammatory responses when exposed to pathogens. Conversely, increasing studies reveal that microbial dysbiosis is associated with many kinds of diseases such as autoimmune and allergic diseases, metabolic diseases, gastrointestinal disorders, and cardiovascular diseases.^10,11^

## 2. The link between hypertension and gut microbiota

In the hypertensive models, notable alterations in the gut microbiota and intestine are reported. The structure and composition of gut microbiota are characterized by increased Firmicutes/Bacteroidetes (F/B) ratio, decreased microbial richness, and reduced abundance of beneficial bacteria.^12^ F/B ratio refers to the ratio of abundances of Firmicutes over Bacteroidetes, which was once considered a measure for microbiome health, but is now less reliable with more inconsistent results reported. Yan et al. found that the abundances of *Klebsiella*, *Clostridium*, *Streptococcus*, *Dysgonomonas*, *Eggerthella*, and *Salmonella* were higher in the fecal samples of hypertensive patients, while the abundances of *Bacteroides*, *Roseburia*, and *Faecalibacterium* were more dominant in healthy individuals.^13^ Li et al.^14^ analyzed the gut microbiota of 41 healthy controls, 56 pre-hypertensive patients, and 99 patients with primary hypertension using metagenomics and metabolomics, and found that the gut microbiota of hypertensive patients had significantly reduced richness and diversity, with changes in microbial composition such as an increased abundance of *Prevotella* and *Klebsiella*. Transfer of the fecal gut microbiota from hypertensive patients to germ-free mice resulted in the blood pressure increase in the recipient mice, indicating a direct microbial influence on blood pressure regulation. Dinakis et a. demonstrated the relationship between gut microbiota and the blood pressure variability. To be specific, diversity of gut microbiota, levels of microbial metabolites, and the bacteria *Lactobacillus* and *Alistipes finegoldii* were associated with lower blood pressure variability whereas *Prevotella* and *Clostridium* with higher blood pressure variability.^15^ Scientists consistently found hypertension-associated microbial alterations compared to the respective normotensive counterparts, although the inconsistency in bacterial species were reported across different studies, which may be likely due to the nature of geographical differences in gut microbiota. Interestingly, a recent study analyzing the sex differences in the association between gut microbiota and blood pressure of 241 patients in Hong Kong, China concluded that dysbiosis was strongly associated with 24-hour ambulatory blood pressure in women but not men.^16^ Higher blood pressure are also reported to be associated with an increase in intestinal permeability, an enlargement of the muscular layer and fibrotic area of the intestinal wall, a decrease in tight junction proteins, and a reduction in the length of the intestinal villi.^17^ These findings highlight the association between hypertension and the structural integrity and functionality of the intestines. These studies demonstrate that gut microbiota imbalance is closely related to hypertension, and different gut microbiota may exhibit different effects on blood pressure.

## 3. Mechanisms linking gut microbiota and hypertension

### 3.1. Gut microbial metabolites and hypertension

**Figure 1.**
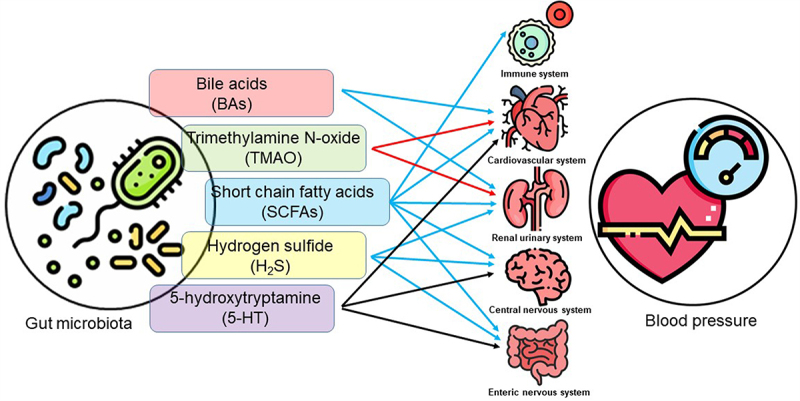
The gut microbiota derived or modified metabolites in the regulation of blood pressure. Five groups of metabolites have diverse effects five major systems to contribute to the blood pressure regulation. Red arrows indicate blood pressure-increasing effect; blue arrows indicate blood pressure-lowering effect; black arrows indicate mixed effects on blood pressure.

Metabolites have been shown to regulate blood pressure. We focus on the SCFAs, trimethylamine-N-oxide (TMAO), BAs, hydrogen sulfide (H_2_S), and 5-hydroxytryptamine (5-HT), all of which have been intensively studied in many research fields. The impacts of these metabolites on blood pressure and currently associated mechanisms are summarized in Figure 1 and Table 1.

**Table 1.** Mechanisms of microbiota-derived metabolites in blood pressure regulation.

Metabolite		Blood pressure change	Mechanisms
Short chain fatty acids	Acetate	Lower	1. Activate Treg cells, enhance mRNA levels of Tjp1.^24^ 2. Reduced expression of IL17a and IL6.^24^
	Propionate	Increase or Lower	1. Activate Olfr78 to raise blood pressure,^30^ while activate Gpr41 to lower blood pressure.^28^ 2. Attenuate T cells responses to Ang II, and reduce Th17 and memory T cells, leading to a decrease in blood pressure.^26^
	Butyrate	Lower	1. Enhance β-oxidation of the intestinal mucosa.^32^ 2. Alleviate immune inflammatory reactions.^32^
Trimethylamine-N-oxide		Increase	1. Enhance the pro-hypertensive effect of Ang II.^36^ 2. Decrease vascular compliance, induce endothelial dysfunction, and atherosclerosis.^38^
Bile acid		Lower	1. Lower succinate, a pro-hypertensive metabolite.^40^ 2. Activate multiple receptors, including FXR, PXR, VDR, and TGR5.^42,43^
H_2_S		Lower	1. Dilate peripheral vessel through K-ATP channels.^58^ 2. Attenuate Ang II-dependent autonomic dysfunction.^59^ 3. Improve endothelial by activating the PPARδ/eNOS pathway.^60^ 4. Inhibit renin synthesis and release.^61^
Serotonin		Increase or Lower	1.Activate vagal nerve and strong vasoconstriction.^62,63^ 2.Collaborate with enteric nervous system and the autonomic nervous system.^64^

#### 3.1.1. Short-chain fatty acids (SCFAs)

SCFAs are produced as a result of fiber fermentation. It mainly includes acetate, propionate, and butyrate, three of which account for 95% of the total SCFAs. SCFAs play an important role in regulating blood pressure and sympathetic nerve activity.^18,19^ Clinical studies have shown a negative correlation between blood pressure levels and SCFA levels in hypertensive patients.^20^ This finding has also been aligned in animal experiments, where the addition of SCFAs to the drinking water of spontaneously hypertensive rats (SHR) prevented further elevation of blood pressure.^18^ In mineralocorticoid excess-treated mice, SCFAs lowered both systolic and diastolic blood pressure. However, Yang et al. reported higher SCFA levels in the cecal contents of SHR, compared to Wistar Kyoto (WKY) rats, likely due to the deficient absorption of SCFAs in the hypertensive rats.^21^ Consistently Cuesta-Zuluaga J et al. found higher fecal SCFA concentrations were associated with hypertension.^22^ Calderón-Pérez L et al. demonstrated that the SCFA levels were significantly higher in feces and lower in plasma in the hypertensive group.^23^ It would be interesting to demonstrate if the accumulation of SCFAs in the feces of hypertensive patients is due to the deficiency in intestinal absorption of SCFAs. Further investigation of the mechanisms has shown that SCFAs regulated blood pressure mainly by the role of G protein-coupled receptors (GPCRs) of SCFAs and their downstream immune and epigenetic effects. Kaye et al.^24^ reported that SCFAs supplementation decreased the blood pressure of saline or angiotensin II (Ang II) induced hypertensive mice. However, this blood pressure-reducing effect wasn’t found in the GPR41, GPR43, GPR109A or GPR43/109A knockout mice, which suggested that these receptors were in the blood pressure regulation of SCFAs. Moreover, acetate intaking changed DNA methylation in regions that activate T regulatory (Treg) cells, enhanced mRNA levels of tight junction protein (Tjp1), and restrained expression of interleukin 17a (IL17a) and interleukin 6 (IL6), the proinflammatory cytokines. Smith et al. demonstrated SCFAs could bind to GPR43 in T cells in large intestine, and trigger T cells into Treg cells by the way of polarization.^25^ Bartolomaeus et al. investigated the effect of propionate supplements on the Ang II-induced hypertensive mice. The result showed that propionate could ameliorate the development of hypertension by reducing blood pressure, and attenuating cardiac hypertrophy, fibrosis, vascular dysfunction, and atherosclerosis. Propionate also mitigated systemic inflammation by reducing splenic effector memory T cell frequencies and splenic T helper 17 (Th17) cells and local cardiac immune cell infiltration. However, the cardioprotective effects were not found in regulatory T cell-depleted Ang II-infused mice, suggesting that regulator T cells took part in the benefit effects of propionate.^26^ On the other hand, SCFAs can bind to GPR41 in vascular endothelial cells and decrease blood pressure by the effect of vasodilation.^27^ SCFAs can also relax mesenteric arteries through the GPR41/GPR43 pathway, attenuating hypertension.^28^ Additionally, SCFAs can protect podocytes on the glomerular basement membrane by acting through GPR109A, improving proteinuria, reducing glomerulosclerosis and tissue inflammation, and ultimately lowering blood pressure.^29^ On the contrary, activation of olfactory receptor 78 (Olfr78) can increase blood pressure through the response of juxtaglomerular cells to SCFAs which promotes cyclic adenosine monophosphate (cAMP) production and increases renin release. The increase in blood pressure is prevented when the Olfr78 gene is knocked out.^30^ Exogenous supplementation of SCFAs and a high-fiber diet can also significantly improve salt-induced hypertension and inhibit myocardial and renal fibrosis by downregulating the RAS and the *Egr1* gene expression in the kidneys.^31^ In addition, β-oxidation of the intestinal mucosa can be accelerated by SCFAs, butyrate in particular, which also deplete oxygen in the intestinal lumen, thus providing a favorable environment for the homeostasis of intestinal microbiota, and ultimately alleviating hypertension and hypertension-related immune inflammatory reactions and target organ damage.^32^

These findings suggest that SCFAs can participate in the control of blood pressure through multiple systems and pathways. Proper knowledge of the role of SCFAs in blood pressure regulation and their specific mechanisms may provide new avenues and directions for the prevention and management of hypertension.

#### 3.1.2. Trimethylamine N-oxide (TMAO)

Trimethylamine (TMA) is a metabolite of choline produced by gut microbiota. Oxidation of TMA in the host liver generates TMAO. Recent research has found a link between TMAO and the increased risk for hypertension. A meta-analysis showed that individuals with high levels of TMAO had a significantly higher incidence of hypertension compared to normal individuals.^33^ Another updated meta-analysis reported that higher circulating TMAO was related to higher systolic blood pressure, but there was no significant association between TMAO and diastolic blood pressure.^34^ Similar findings were observed in rats where high TMAO levels in SHR plasma increased plasma osmotic pressure, increasing renal water reabsorption and circulating blood volume, potentially leading to hypertension.^35^ Studies in rats showed that injecting TMAO did not affect blood pressure, but concurrent injection of Ang II and TMAO prolonged the increase in blood pressure.^36^ On the other hand, the administration of TMA inhibitor, 3,3-dimethyl-1-butanol, can lower TMAO levels in circulation and ultimately inhibit TMAO’s prolongation of the hypertensive effect of Ang II.^37,38^ Therefore, TMAO impacts blood pressure in an indirect manner by enhancing the pro-hypertensive effect of Ang II.^45^

#### 3.1.3. Bile acids (BAs)

Primary BAs are synthesized in the liver from cholesterol. Additionally, the primary BAs are conjugated with glycine or taurine for better solubility. Gut microbiota possess bile salts hydrolases and dehydroxylases which can deconjugate glycine/taurine and convert primary bile acids to secondary bile acids, respectively.^39^ Chakraborty et al. demonstrated an altered profile of gut microbiota with a higher abundance of deconjugating microbes and lower levels of conjugated BAs, and the administration of taurine and taurocholic acid was sufficient to lower the blood pressure of hypertensive rats.^40^ Conjugated BAs exhibited antihypertensive effects, presumably due to their capability to lower succinate, a proven pro-hypertensive metabolite.^40^ Cholic acid and its taurine conjugate can increase the activity of large-conductance calcium-activated potassium channels in smooth muscle cells, leading to vasodilation.^41^ In terms of the mechanisms, multiple receptors of BAs have been demonstrated up to date, including pregnane X receptor, farnesoid X receptor (FXR), vitamin D receptor, and G protein-coupled receptor Gpbar1 (TGR5) pathways.^42,43^ Among these, TGR5 and FXR are commonly activated by both primary (i.e. cholic acid, chenodeoxycholic acid) and secondary BAs (i.e. deoxycholic acid, lithocholic acid).^44^ BAs can increase intracellular calcium concentration and NO production in endothelial cells and smooth muscle cells via the activation of Akt or the regulation of cystathionine-γ-lyase expression and activity, both of which are dependent on TGR5, to induce vasodilation.^52^ In a recent study, Shi et al. reported that intermittent fasting lowered blood pressure in SHR stroke-prone rats with a reshaped BA profile with significantly reduced cholic acid. Supplementation of cholic acid was found to lower blood pressure in the SHR stroke-prone rats. Additionally, a TGR5 agonist, oleanolic acid, was found to improve vascular function and lower blood pressure.^46^ Furthermore, activation of FXR by BAs upregulated Ang II type 2 receptor, which can prevent the development of salt-sensitive hypertension in rats when bound to an agonist, suggesting that BA-induced FXR activation may be a potential target for treating salt-sensitive hypertension.^47,48^ TGR5 agonist BA activated receptor 501 and FXR agonist PX20606, have shown to alleviate portal hypertension by reducing inflammation and promoting vasodilation.^49,50^ Therefore, BAs may become a new target for future research, especially for understanding the causes of salt-sensitive hypertension and finding corresponding treatment methods.

#### 3.1.4. H_2_S

H_2_S is an important gas mediator and signaling molecule in the human body, acting as a reducing agent. It is abundant in the colon, primarily produced by enzymatic reactions from intestinal epithelial cells and gut microbiota, and can participate in regulating arterial blood pressure. It has been shown that diet restriction, dietary protein, and edible plants all increase H_2_S levels in the feces,^51–53^ H_2_S plays a crucial role as a biological mediator in the circulatory system, participating in numerous biological signaling pathways within the body, including the regulation of blood pressure and cardiac protection. H_2_S derived from the colon can target blood vessels when absorbed into the intestinal blood vessel, stimulate sensory fibers of the intestinal nervous system, and feedback to the central nervous system to control the circulatory system, ultimately participating in blood pressure regulation.^54^ Szlezak et al. found that the expression of cystathionine γ-lyase involved in H_2_S metabolism in SHR liver was significantly lower than that of WKY, which indirectly indicated that H_2_S was related to hypertension.^55^ Mice deficient in cystathionine γ-lyase develop hypertension at the age of 8 weeks, and injection of exogenous H_2_S rescued the mice from high blood pressure.^56^ Huc et al. found that injection of H_2_S into the rat colon lowered arterial blood pressure while increasing portal vein pressure, suggesting that colon-derived H_2_S may contribute to the regulation of portal vein pressure and systemic arterial blood pressure.^57^ Similarly, Tomasova et al. found that injecting H_2_S donor Na_2_S into the colon produced a similar blood pressure downregulation effect, in a dose-dependent manner. In this study, H_2_S-induced reduction in blood pressure in the SHR may be attributed to the peripheral vasodilation mediated by K-ATP channels.^58^ Long-term intraventricular infusion of NaHS, an H_2_S donor, can attenuate Ang II-induced increase and activation of paraventricular nucleus astrocytes, relieve Ang II-dependent autonomic dysfunction, and reduce blood pressure.^29^ Xiao et al. also found that H_2_S improved endothelial dysfunction in hypertensive patients and rats with renovascular hypertension by activating the PPARδ/eNOS pathway, thereby attenuating hypertension.^60^ In addition, H_2_S can improve renovascular hypertension by inhibiting the synthesis and release of renin.^61^ Taken together, this suggests that colonic-derived H_2_S may contribute to blood pressure regulation.

#### 3.1.5. 5-hydroxytryptamine (5-HT)

80%-90% of the body’s 5-HT is produced by specialized enterochromaffin cells in the intestine. Studies have found that the colon-derived 5-HT biosynthesis is modulated by spore-forming bacteria.^62^ Butyrate, cholate, propionate, tyramine and deoxycholate were reported to stimulate the secretion of 5-HT from RIN14B cells (a rat islet cell line). The subsequent study showed that deoxycholate increased serum 5-HT levels in germ-free mice.^62^ 5-HT released into the bloodstream can bind to its receptors, which can stimulate the activity of the vagal nerve in the gut and cause strong vasoconstriction in the vascular system. However, if sympathetic nervous system activity increases or vagal nerve excitability decreases, 5-HT’s regulatory role in the brain’s cardiac control area weakens, leading to an increase in blood pressure.^63^ 5-HT can collaborate with the enteric nervous system and the autonomic nervous system to control gut motility, and the autonomic nervous system, on the other hand, can change activity and blood pressure by integrating inputs from the peripheral and central nervous systems and gut microbiota metabolites.^64^ In contrast, 5-HT can lower blood pressure in vivo depending on its binding to different 5-HT receptors. Regarding the complex role of 5-HT in hypertension and normotension, Watts et al. have composed excellent comprehensive reviews on 5-HT in blood pressure regulation.^65,66^

### 3.2. High-salt diet-induced gut dysbiosis affects hypertension

Approximately 30% of hypertension prevalence can be attributed to high-salt diet.^67^ Mozaffarian et al. conducted a comprehensive analysis of extensive data, revealing that sodium intake can elevate blood pressure in both normal individuals and hypertensive patients.^68^ The mechanisms of hypertension induced by high-salt intake include change of sodium-water homeostasis, vascular tone, and immune cell homeostasis.^69^ Recent studies indicate that gut microbiota is involved in the pathogenesis. Compared with a normal diet group, it was found that high salt intake changed the composition of gut microbiota and their circulating metabolites,^70^ increased the F/B ratio, promoted inflammatory responses and induced the occurrence of hypertension. Bier et al. identified the fecal microbiota of normal and high-salt-induced hypertensive rats, it was also found that high salt intake altered the gut microbiota composition and metabolites.^71^ A randomized controlled intervention study involving 145 untreated hypertensive patients showed that a low-salt diet increased SCFAs in circulation, which in turn lowered blood pressure and improved vascular compliance.^20^ Furthermore, Ferguson et al. demonstrated that high salt-induced gut microbiota predisposed germ-free mice to intestinal inflammation and hypertension, and overactivation of dendritic cells may be involved in this process.^72^ Wilck et al. found that feeding a high-salt diet to mice depleted the intestinal *Lactobacillus*, the reduction of which induced Th17 cells, drove the immune response, and aggravated encephalitis and hypertension.^73^ High salt-induced Th17 cells released interleukin-17, interleukin-1β and TNF-α jointly promote the reabsorption of sodium ions by the renal tubules.^74^ This led to the retention of water and sodium in the body, further causing hypertension.

## 4. Gut microbiota intervention in hypertension treatment

### 4.1. Gut microbiota and antihypertensive medications

Individual disparity in drug response has been documented. Gut microbiota is recently proposed to be involved in the modulation of drug efficacy. Growing evidence has shown that gut microbiota has a similar metabolic capability as the liver.^75,76^ Numerous gut microbiota possess a diverse of enzymatic activities (oxidation, reduction, hydrolysis dicarboxylic reaction, *etc*.) which are significant to the drug metabolism.^77^ Additionally, the metabolites of microbiota can also influent liver enzymes, such as cytochrome P450 (P450) activities.^78^ All the processes and reactions conducted by the gut microbiota can unpredictably affect drug absorption, distribution, and metabolism.^79^ Conversely, drugs entering the body may also alter the composition of gut microbiota, with significant effects on its structure and metabolic capacity.^80^ Therefore, drug biotransformation and gut microbiota have mutual influences. Gut microbiota can metabolize drugs, affecting their bioavailability and toxicity; on the other hand, drugs can alter the structure and composition of gut microbiota, affecting its nutritional and metabolic status.^81^

As for hypertension, the following section will discuss the interactions between gut microbiota and the main antihypertensive drugs: calcium channel blockers, angiotensin-converting enzyme inhibitors (ACEis), Ang II receptor blockers (ARBs), diuretics, and beta blockers.

#### 4.1.1. Calcium channel blockers

Amlodipine undergoes hepatic enzyme-mediated metabolism in the human body, resulting in the formation of pyridine metabolites. These metabolites undergo further processes, including oxidative deamination, deesterification, and hydroxylation of the fatty group.^82^ A recent study employed amlodipine compound to incubate with stool from both humans and rats. The authors demonstrated a gradual escalation in the concentration of the pyridine metabolite with the extension of the incubation period. An analogous reduction in the residual amlodipine concentration occurred concurrently. The metabolism of amlodipine was greatly reduced when fecal material from the rats that were treated with antibiotics was used in the in vitro culture with amlodipine. This indicates that amlodipine’s pharmacokinetics may be influenced by the gastrointestinal microbiota via its potential involvement in its biotransformation. Co-administration of antibacterial drugs and amlodipine found that oral ampicillin could increase the bioavailability of amlodipine. Therefore, antibiotics depleted gut microbiota, reduced the metabolism of amlodipine, and ultimately increased its bioavailability.^83^

#### 4.1.2. ACEis

Besides the role in renin angiotensin system, ACEis reduced the muscular and fibrotic portions of the intestinal wall in hypertension and increased the length of the intestinal villi. This suggests that the process of ACEi-mediated blood pressure reduction was related to the restoration of the pathological state of the intestine in hypertension.^84^ So far, many ACEis have been shown the association with gut microbiota, including enalapril, quinapril, lisinopril, captopril, and benazepril. Studies have shown that the plasma level of TMAO in SHR rats is significantly higher than that in WKY rats. However, treatment with enalapril significantly reduced the plasma TMAO level.^85^ Konop et al. reported that treatment with enalapril lowered the plasma level of TMAO with a trend toward higher 24 h urine excretion of TMA and TMAO but no significant difference in gut microbiota composition.^86^ For captopril, Santisteban et al. reported that captopril improves pathological changes in the gut.^84^ These changes were found to be independent of captopril intake but associated with altered gut microbiota and dampened posterior pituitary neuronal activity.^87^ Additionally, captopril-mediated beneficial changes in the gut brain axis are transgenerational.^88^ Conversely, SHRs with depleted gut microbiota responded with a greater blood pressure reduction to captopril.^89^ Furthermore, *Coprococcus comes* was recently identified as a gut microbe that compromises the effectiveness of ACEi with an ester structure.^90^ Thus, these suggest that gut microbiota may be a culprit for resistant hypertension. The metabolic effects of gut microbiota on resistance to antihypertensive drugs have been reviewed elsewhere.^77^

#### 4.1.3. ARBs

The antihypertensive effect of ARBs losartan is related to the gut microbiota. Robles-Vera et al.^91^ found that losartan treatment improved dysbiosis. Specifically, losartan restored the F/B ratio in SHR gut to a level similar to that in WKY rats and increased the number of bacteria producing acetate and propionate. In addition, losartan improved intestinal barrier integrity and reduced sympathetic drive. Fecal matter transfer from losartan-treated SHR to SHR reduced blood pressure. Thus, the protection of blood vessels and reduction in blood pressure may be partially attributed to losartan-induced changes in gut microbiota.^91^ Of late, another recent study declared a similar result.^92^ Namely, in addition to blood pressure lowering, losartan decreased the abundances of *Ruminococcaceae*, *Streptococcus*, *Turicibacter*, and the F/B ratio, increased the abundances of *Alistipes*, *Bacteroides*, and *Butyricimonas* in SHR. Additionally, losartan decreased the abundance of lactate-producing bacteria while increasing the abundance of Bifidobacterium and SCFAs-producing bacteria. All the results showed that losartan could change the characteristics of the gut microbiota in hypertension and rebalance the gut microbiota, which may be related to blood pressure lowering. Conversely, several ARBs, including losartan, were demonstrated to be transformed by the gut microbiota in an *in vitro* assay.^93^

#### 4.1.4. Diuretics

Spironolactone, a mineralocorticoid receptor antagonist, was demonstrated to restore F/B ratio and population of acetate-producing bacteria in SHR to WKY level.^94^ Spironolactone was also reported in this study to lower the Th17 cells proportion in mesenteric lymph nodes and Th17 infiltration in aorta, improve aortic endothelial function and reduce systolic blood pressure. In an in-silico study, two sulfonamide diuretics, hydrochlorothiazide and indapamide, were found to bind the Nicotinamide adenine dinucleotide phosphate (NADPH) binding region of bacterial dihydrofolate reductase and possess antibiotic activity and thereby have the potential to modulate the gut microbiome.^95^

#### 4.1.5. Beta blockers

Metoprolol is primarily metabolized by hepatic cytochrome 2D6, which involves the metabolism of a multitude of endogenous hormones and about 25% of exogenous drugs. Brocker et al. demonstrated patients treated with metoprolol had elevated levels of hippuric acid, hydroxyhippuric acid, and methyluric acid, all of which are gut microbiota-dependent metabolites.^96^ These results suggested either a potential alteration in gut microbial species or merely a change in microbial transcriptomics. Nonetheless, these data indicate that long-term treatment with metoprolol may affect the metabolic functions of the gastrointestinal microbiota. Alexandra et al. tested 1135 stool samples by metagenomic analyses, and they reported relations between gut microbiome and 19 drug groups, including beta blockers and ACEis.^97^ Lim *et al*. administered a combination therapy of propranolol (nonselective beta blocker) with rifaximin (a gastrointestinal-selective antibiotic) in male human patients with advanced cirrhosis.^98^ The results showed significant improvements of the hepatic venous pressure gradient (HVPG) response and a reduction of bacterial translocation-related markers. Rifaximin demonstrated an additive effect in diminishing HVPG by inhibiting bacterial translocation more effectively than monotherapy with conventional propranolol. Unfortunately, no microbiota analysis was performed to document any changes in gut microbes.

### 4.2. Antibiotics and hypertension

Antibiotics can change gut microbiota and affect hypertension. Current knowledge of antibiotics on blood pressure of certain hosts and gut microbiota is summarized in Table 2. Honour et al. first reported that oral administration of neomycin attenuated the hypertension in corticosterone-induced hypertensive male Sprague-Dawley rats.^99^ Yang et al. reported oral minocycline, a broad-spectrum antibiotic freely crossing the blood-brain barrier, not only reduced blood pressure, but also rebalanced the gut microbiota in the chronic Ang II infusion rat model.^100^ Galla et al. showed that amoxicillin remodeled the structure of gut microbiota in the early life of Dahl salt-sensitive rats (*e.g*. reduction in *Veillonellaceae*, a family of bacterium with succinate-producing capacity) and decreased blood pressure.^101^ Reversely, Hsu et al. found that exposure to minocycline could also increase blood pressure in experimental Sprague-Dawley rats on a high fructose diet, associated with a higher F/B ratio and a lower abundance of *Lactobacillus* and *Ruminococcus*.^102^ Galla et al.^103^ demonstrated that oral administration of minocycline and vancomycin, but not neomycin, lowered systolic blood pressure in the SHR, while all these three antibiotics increased systolic blood pressure of the Dahl salt-sensitive rat. The result suggested that the antihypertensive effects of different antibiotics vary between animal models. Several factors that may contribute to such differences include the initial gut microbiota prior to antibiotics treatment (which are determined by host genome, bedding materials, diets, etc.), targeted gut microbiota of the antibiotics (e.g. Gram positive, Gram negative), environmental factors during treatment (e.g. diets, stress).

**Table 2.** Effectiveness of antibiotics and the associated microbiota in different experimental animals.

Antibiotic	Object of study	Blood pressure change	Gut microbial change	Reference
Minocycline	Ang II-infused Sprague-Dawley rats	Lower	↑*Akkermansia*, *Bacteroides*, *Enterorhabdus*, and *Marvinbryantia*	^100^
	high fructose diet-induced Sprague-Dawley rats	Increase	↑*Akkermansia* ↓*Lactobacillus*, *Ruminococcus*, and *Odoribacte*	^102^
	Dahl salt‐sensitive rats	Increase	↑*Firmicutes, Proteobacteria*, and *Verrucomicrobia*, ↓*Actinobacteria, Bacteroidetes, Cyanobacteria, Deferribacteres, TM7*, and *Tenericutes*	^101^
	SHR	Lower	↑*Actinobacteria, Cyanobacteria, Deferribacteres*, and *Firmicutes* ↓*Bacteroidetes, Proteobacteria, TM7*, and *Tenericutes*	^101^
Vancomycin	Dahl salt‐sensitive rats	Increase	↑*Bacteroidetes, Cyanobacteria, Elusimicrobia, Fusobacteria, Proteobacteria*, and *Verrucomicrobia* ↓*Actinobacteria, Deferribacteres, Firmicutes, TM7*, and *Tenericutes*	^101^
	SHR	Lower	↑*Bacteroidetes, Cyanobacteria, Proteobacteria*, and *Verrucomicrobia*, ↓*Firmicutes, TM7*, and *Tenericutes*	^101^
Neomycin	Dahl salt‐sensitive rats	Increase	↑*Bacteroidetes, Cyanobacteria, Fusobacteria*, and *Verrucomicrobia*, ↓*Actinobacteria, Deferribacteres, Firmicutes, Proteobacteria, TM7*, and *Tenericutes*	^101^
	SHR	No change	↑*Bacteroidetes, Cyanobacteria, Elusimicrobia*, and *Verrucomicrobia* ↓*Firmicutes, Proteobacteria, TM7*, and *Tenericutes*	^101^
	Corticosterone-induced Sprague-Dawley rats	Lower	Not mentioned.	^99^
Amoxicillin	Dahl salt‐sensitive rats	Lower	↓*Ruminococcus*, *Oscillospira*, *Anaerovibrio*, *Lactobacillus, Pseudomonas*, *Coprococcus*, *Dorea*, *Clostridium, Roseburia*, *Turicibacter*, and *Prevotella* ↑*Blautia*, *Prevotella*, *Enterobacter, Enterococcus, Klebsiella*, *Sutterella*, and *Bacteroides*	^101^

### 4.3. Gut microbiota-targeted intervention and its influence on hypertension

#### 4.3.1. Probiotic supplementation

Probiotics are a collective term for living microorganisms that are of specific health benefits and biological activity. Epidemiological data analysis has shown that probiotics can lower blood pressure in hypertensive patients and have a moderate effect on blood pressure in healthy populations,^104–106^ In experimental animals, the antihypertensive mechanisms of probiotics were widely studied. Kong et al. found that supplementing yogurt with probiotics in SHR significantly reduced blood pressure, altered gut microbiota composition, and increased the abundance of SCFA-producing bacteria and SCFA levels (acetate, propionate, butyrate, and valerate) in feces to regulate gut microbiota.^107^ Robles-Vera et al. reported chronic treatment with *Bifidobacterium breve* and *Lactobacillus fermentum* prevented blood pressure increase and restored gut dysbiosis (*e.g*. reduced F/B ratio, increased butyrate-producing bacteria).^18^ The treatment also normalized endotoxemia and restored the Th17/Treg balance in mesenteric lymph nodes. Moreover, besides beneficial impacts on blood pressure, the intervention with probiotics can significantly decrease the amount of gram-negative *Proteobacteria* and gram-positive *Clostridium*, inhibit the increase of NADPH oxidase activity and reduce the expression of neuronal nitric oxide synthase (NOS) gene, reduce NADPH oxidase-driven reactive oxygen species production, and prevented the impairment of endothelium function.^108–110^

#### 4.3.2. High-fiber diet

Dietary intervention to modulate gut microbiota structure could be an innovative nutritional therapy for hypertension. It was reported that increased dietary fiber from oat bran reduced the use of antihypertensive drugs, lowered office and 24 h ambulatory blood pressure, while changed β diversity and increased the relative abundance of *Bifidobacterium* and *Spirillum*.^111^ Marques et al. treated mice with a high-fiber diet or a diet supplemented with acetate, leading to changes in gut microbiota and increased levels of SCFAs, which had a protective effect on hypertension and heart failure.^31^ These findings suggest that dietary high-fiber interventions may lower blood pressure by improving gut microbiota diversity and abundance. Recently, a phase II randomized clinical trial reported that administration of prebiotic acetylated and butyrylated high-amylose maize starch for 3 weeks reduced 24-hour systolic blood pressure in treatment-naïve hypertensive patients, Importantly, the blood pressure reduction is independent of age, sex, and body mass index. Despite of relatively small sample size (*n* = 20), this study revealed promising use of prebiotics for hypertension management.^112^

#### 4.3.3. Fecal microbiota transplantation intervention

Fecal microbiota transplantation is a potential therapeutic intervention aimed at modulating gut microbiota by introducing the fecal contents of healthy donors into the gastrointestinal tract of patients to re-construct the gut microbiota. However, the use of fecal microbiota transplantation is currently limited because of the difficulties in quality control of so-called healthy microbiota.^113^ Washed Microbiota Transplantation (WMT) sanitizes the feces by removing food remnants, parasitic eggs, viruses, and inflammatory factors from fecal matter, which provides a relatively safe and quality-controllable microbiota for transplantation.^114^ In a clinical study to evaluate the impacts of WMT on blood pressure, 260 volunteers (73 with hypertension and 187 with normotension) were initially recruited for two WMTs in two separate visits. Among these, 72 subjects (19 with hypertension and 53 with normotension) finished two WMTs, while the others only received one WMT. The authors reported several findings: (1) one WMT significantly lowered blood pressure upon hospital discharge in comparison to admission; (2) one extra WMT did not lower blood pressure more; (3) WMT lowered blood pressure in the hypertensive subjects without intake of antihypertensive medications; (4) intra-anal administration seems more efficient in lowering blood pressure than oral administration; (5) 3.7% side effects were reported, which mainly included abdominal pain, diarrhea and dizziness.^115^ Although beneficial effects were observed, one limitation was that the preparation of washed microbiota was done aerobically. This would eliminate a significant amount of bacteria. One may wonder if the blood pressure-lowering effects may be better when efforts to preserve the viability of anaerobic microbiota are made. Further research is needed to evaluate other physiological parameters in addition to blood pressure, which would provide evidence for any potential major side effects.

## 5. Machine learning-based gut microbiota analysis in hypertension

In the past decade, scientists have generated a large volume of microbiota data. To further exploit the data, different machine learning methods were applied in the analysis to facilitate the interpretation of microbiota data from a multiple-dimensional perspective. In contrast to the statistical comparisons, machine learning methods can conduct multilayered calculations to conclude the relationships among variables for automatic pattern discovery. The discovered pattern can be further used for sample classification, sample characterization, biomarker identification, and association studies. Several machine learning algorithms are commonly used in the analysis, including random forest, neural network, decision tree, *etc*. Due to the complexity of propounding factors that impact the results, it is recommended to evaluate more than one method and conclude with the method that exhibits the best performance. Indeed, machine learning has been tested for the prediction and diagnosis of multiple diseases such as breast cancer,^116^ and inflammatory bowel diseases,^117^ which are related to gut microbial alterations. In the gut microbiota and hypertension study, research efforts were exploratory at best with great potential. Islam et al. reported the use of machine learning in a dataset of three South Asian countries to successfully predict hypertension and age and body mass index being the top associated factors.^118^ In another study by Aryal et al., the authors randomly analyzed the American Gut Project data from 478 subjects with cardiovascular diseases and 473 subjects with non-cardiovascular diseases.^119^ With the top 25 operational taxonomic unit features, the area under the receiver operating characteristic curve was 0.7 in the random forest algorithm. Despite the rapid progress of machine learning in this field, there remains a research gap along the way. One major roadblock to overcome is “precision”. The precision is not only about the accuracy of the prediction results, but also about the accuracy of the input data provided. Gut microbiota is such a complex ecosystem that can be tremendously influenced by many environmental, host and endogenous factors. Inclusion of all relevant parameters (i.e. age, sex, medication history) of the host for adjustment could greatly accurize the prediction. Nonetheless, gut microbiota-based supervised machine learning modeling has proven to be a promising and innovative approach for the diagnostic screening of hypertension, notwithstanding its nascent stage.

## 6. Conclusion

With the rapid advances in sequencing techniques in the past two decades, gut microbiota has been extensively studied for its role in health and diseases. The research focusing on the role of bacteria in blood pressure regulation has evolved simultaneously. Our review summarized the current knowledge on 1) the mechanisms of gut microbiota for high blood pressure; 2) the interactions between gut microbiota and antihypertensive drugs for blood pressure regulation; 3) the potential application of machine learning in the prediction and diagnosis of hypertension. Despite great potential, scientists are facing challenges in reproducibility, and research rigor in the microbiota study. To combat these challenges, a standardized protocol for microbiota study from sample collection to data presentation was timely developed.^120^ Additionally, future studies on the microbiota may consider several important variables, including circadian rhythm^121^ and sex differences,^16,122^ both of which have been closely linked to microbiota patterns. Taken together, although the issues exist, the development of research techniques and protocols will facilitate overcoming such challenges.

## Data Availability

Data sharing not applicable – no new data generated.
